# Sec16A, a key protein in COPII vesicle formation, regulates the stability and localization of the novel ubiquitin ligase RNF183

**DOI:** 10.1371/journal.pone.0190407

**Published:** 2018-01-04

**Authors:** Yan Wu, Xiao Peng Guo, Soshi Kanemoto, Yujiro Maeoka, Atsushi Saito, Rie Asada, Koji Matsuhisa, Yosuke Ohtake, Kazunori Imaizumi, Masayuki Kaneko

**Affiliations:** 1 Department of Biochemistry, Institute of Biomedical and Health Sciences, Hiroshima University, Hiroshima, Japan; 2 Department of Nephrology, Hiroshima University Hospital, Hiroshima, Japan; 3 Department of Stress Protein Processing, Institute of Biomedical and Health Sciences, Hiroshima University, Hiroshima, Japan; Medizinische Fakultat der RWTH Aachen, GERMANY

## Abstract

We identified 37 ubiquitin ligases containing RING-finger and transmembrane domains. Of these, we found that RNF183 is abundantly expressed in the kidney. RNF183 predominantly localizes to the endoplasmic reticulum (ER), Golgi, and lysosome. We identified Sec16A, which is involved in coat protein complex II vesicle formation, as an RNF183-interacting protein. RNF183 colocalized with Sec16A and interacted through the central conserved domain (CCD) of Sec16A. Although Sec16A is not a substrate for RNF183, RNF183 was more rapidly degraded by the ER-associated degradation (ERAD) in the absence of Sec16A. Sec16A also stabilized the interacting ubiquitin ligase RNF152, which localizes to the lysosome and has structural similarity with RNF183. These results suggest that Sec16A appears to regulate the protein stability and localization of lysosomal ubiquitin ligases.

## Introduction

Proteins in the endoplasmic reticulum (ER) lumen and membrane are subject to ER-associated degradation (ERAD) in the cytoplasm by the ubiquitin-proteasome system [1, 2]. In this process, target proteins are excreted to the cytoplasm through an ER membrane channel and subsequently ubiquitinated by a set of enzymes, including ubiquitin-activating enzyme (E1), ubiquitin-conjugating enzyme (E2), and ubiquitin ligase (E3). The resulting poly-ubiquitinated proteins are subsequently degraded by the 26S proteasome.

Of the three above-mentioned enzymes, E3 is the most crucial, as it participates in the regulation of the substrate preference and degradation rate. Accordingly, we previously searched the human genome to identify ubiquitin ligases involved in ERAD that met the following criteria: 1) a RING-finger domain, which is required for binding with E2 ubiquitin-conjugating enzymes and 2) a transmembrane domain, which is required for ER membrane targeting. As reported, we identified 37 types of transmembrane domain-containing ubiquitin ligases [3]. We further investigated the tissue distributions of these 37 ubiquitin ligases and, consequently, identified the novel kidney-specific ubiquitin ligase RNF183. Moreover, we identified the RNF183-interacting protein Sec16A, which is involved in the formation of coat protein complex II (COPII) vesicles. COPII-coated vesicles play an important role in the membrane transport of folded proteins from the ER to the Golgi [4]. These are formed from sub-complexes, including Sec23/Sec24 and Sec13/Sec31, through a process controlled by Sar1, Sec12, and Sec16A.

In the present study, we aimed to characterize the features of the above-mentioned novel transmembrane ubiquitin ligase RNF183 and elucidate the functional significance of the interaction between RNF183 and Sec16A. In particular, we addressed whether Sec16A, which is not a substrate for RNF183, affects the stabilization and localization of RNF183.

## Materials and methods

### Antibodies

Antibodies against the following proteins were used in this research: β-actin (AC-15, mouse monoclonal; Sigma-Aldrich, St. Louis, MO, USA); Calnexin (C5C9, rabbit monoclonal; Cell Signaling Technology, Danvers, MA, USA); EEA1 (C45B10, rabbit monoclonal; Cell Signaling Technology); GM130 (D6B1, rabbit monoclonal; Cell Signaling Technology); HA-Tag (C29F4, rabbit monoclonal; Cell Signaling Technology); LAMP1 (D2D11, rabbit monoclonal; Cell Signaling Technology); Myc (My3, mouse monoclonal; MBL, Woburn, MA, USA); poly-ubiquitin (FK-2, mouse monoclonal; Nippon Biotest Laboratories, Tokyo, Japan); Sec16A (HPA005684, rabbit polyclonal; Sigma-Aldrich); and V5 (mouse monoclonal; Invitrogen, Carlsbad, CA, USA).

### Cell culture

HEK293 human embryonic kidney cells and HeLa cells were maintained in Dulbecco’s Modified Eagle’s Medium (DMEM; Gibco, Grand Island, NY, USA) supplemented with 10% (v/v) heat-inactivated fetal bovine serum (FBS) at 37°C in a 5% CO_2_, 95% humidified air atmosphere.

### Expression vectors and stable cell lines

Mouse RNF183 and RNF152 constructs, with or without a stop codon, were cloned into the pENTR-D-TOPO vector (Invitrogen). For stable V5-tagged protein expression, the entry clone product and pENTR5’/CMVp vector (Invitrogen) were recombined into the pLenti 6.4/R4R2/V5-DEST vector (Invitrogen) using LR Clonase II Plus enzyme mix (Invitrogen). To produce lentivirus particles expressing V5-tagged mouse RNF183 or RNF152, the pLenti-based expression vector and ViraPower Packaging mix (Invitrogen) were cotransfected into the 293FT cell line. Virus-containing supernatants were harvested, and viral particles were transduced into HEK293 cells. Cells exhibiting stable RNF183 or RNF152 expression were selected using 5 μg/ml blasticidin S hydrochloride (Wako Pure Chemical Industries, Osaka, Japan). The HRD1 stable cell line has been described previously [5].

Human Sec16A sequence was cloned into pENTR-3C Dual Selection Vector (Invitrogen) as an entry clone. For the expression of N-terminal EmGFP-tag, the entry clone product was recombined into Vivid Colors pcDNA6.2/EmGFP-DEST Gateway Vector (Invitrogen) using LR recombination reaction. Sec16A deletion domain constructs were generated by inverse PCR using the entry clone product.

### Reverse transcription-polymerase chain reaction (RT-PCR)

Total RNA profiles of human and murine tissues have been described previously [3]. In this study, 2 μg each of RNAs from human and murine tissues were reverse-transcribed using ReverTra Ace (TOYOBO, Osaka, Japan) and Random Primer (25 pmol; TaKaRa Bio, Shiga, Japan) according to the manufacturers’ protocols. Subsequently, 2 μl of cDNA was amplified in a 20-μl reaction mixture containing each primer (0.2 μM), dNTPs (0.2 mM), Paq5000 DNA polymerase (1 unit; Agilent Technologies, Santa Clara, CA, USA), and 10× PCR buffer. The PCR conditions were as follows: 94°C for 2 min, 18–30 cycles at 98°C for 10 sec, 55.0°C–61.7°C for 30 sec, 72°C for 30 sec, and 72°C for 3 min. Primer sequences, annealing temperatures, and numbers of cycles are listed in S1 Table. PCR products were resolved by electrophoresis on a 2% agarose gel.

### Cycloheximide assay

HEK293 cells with stable expressions of RNF183, RNF152, or HRD1 were transfected with short interfering RNA (siRNA) against Sec16A (CCAGGUGUUUAAGUUCAUCUA) using ScreenFect siRNA (Wako Pure Chemical Industries). At 44 h post-transfection, cells were incubated with 30 μg/ml cycloheximide (Wako Pure Chemical Industries) and 10 μM MG-132 (Wako Pure Chemical Industries) for 0, 1, 2, or 4 h and were subsequently harvested. Proteins were extracted using lysis buffer [20 mM Tris-HCl (pH 7.5), 150 mM NaCl, 10% glycerol, 1% Triton X-100, 100 μM MG-132, Protease inhibitor cocktail Set V (Wako Pure Chemical Industries)]. The lysates were boiled with Laemmli SDS-PAGE sample buffer and subjected to Western blotting using a WSE-6100 LuminoGraph (ATTO Corporation, Tokyo, Japan).

### *In vitro* ubiquitination assay

An *in vitro* ubiquitination assay was performed as described previously [3]. V5-tagged RNF183 protein was produced using the T_N_T Quick coupled transcription/translation system (Promega Corporation, Madison, WI, USA). Reaction products were immunoprecipitated with a V5-specific antibody and mixed with a recombinant rabbit ubiquitin-activating enzyme (E1, 100 ng), GST-UbcH5c (E2, 100 ng), and HA-Ubiquitin (10 μg; all purchased from Boston Biochem, Cambridge, MA, USA) in a 100 μl volume of reaction buffer containing 40 mM Tris-HCl (pH 7.5), 5 mM MgCl_2_, 2 mM ATP, and 2 mM dithiothreitol. The reaction solution was incubated at 30°C for 90 min and immunoprecipitated with the anti-V5 antibody. Immunoprecipitates were subjected to Western blotting using anti-HA and anti-V5 antibodies.

### Immunocytochemistry

HeLa cells stably expressing V5-tagged RNF183 were grown on coverslips, fixed with 4% paraformaldehyde for 15 min, and permeabilized with methanol for 10 min at -20°C, followed by blocking with 5% normal goat serum for 60 min. Cells were labeled overnight at 4°C with an anti-V5 antibody to detect V5-tagged RNF183, as well as antibodies specific for organelle markers; subsequently, cells were incubated with an Alexa Fluor 488- or 568-conjugated goat anti-mouse or anti-rabbit IgG (H+L) secondary antibody (Invitrogen), respectively, for 60 min at room temperature. ProLong Diamond Antifade Mountant with DAPI (Invitrogen) was used to mount coverslips on the slides. Fluorescence images were acquired using a FluoView FV1000 (Olympus Corporation, Tokyo, Japan).

### Immunoprecipitation

HEK293 cells exhibiting stable expression of mouse RNF183 were lysed in lysis buffer [20 mM Tris-HCl (pH 7.5), 150 mM NaCl, 10% glycerol, 1% Triton X-100, 100 μM MG-132, Protease inhibitor cocktail] for 20 min. Supernatants were incubated with anti-V5 antibody at 4°C for 1 h, followed by incubation with Protein G Agarose Beads (Invitrogen) for 1 h; subsequently, the beads were rinsed three times with a wash buffer [20 mM Tris-HCl (pH 7.5), 150 mM NaCl, 10% glycerol, 0.1% Triton X-100]. Immunoprecipitates were boiled with Laemmli SDS-PAGE sample buffer and analyzed by Western blotting.

### Proteome analysis

RNF183 precipitates obtained as described in the previous section were used as the proteome analysis sample. Precipitate-bound beads were suspended in 25.5 μl of bicarbonate ammonium (21.25 mM, Wako Pure Chemical Industries). After adding 1.5 μl of dithiothreitol (12.5 mM, Thermo Scientific), the mixture was incubated at 95°C for 5 min. After cooling to room temperature, 3 μl of iodoacetamide (25 mM, Wako Pure Chemical Industries) were added, and the mixture was incubated at room temperature for 20 min. Next, 10 μl of 30 ng/μl trypsin (Promega Corporation) were added, and the mixture was incubated at 37°C for 3 h. Another 10 μl of trypsin were added, and the mixture was incubated overnight at 30°C. Finally, 2.5 μl of trifluoroacetate (Sigma-Aldrich) were added to terminate the reaction, and the sample was desalted using a C18 Spin Column (Thermo Fisher Scientific), followed by concentration for 2 h on a SpeedVac. Concentrates were analyzed on a TripleTOF 5600+ System with Eksigent nanoLC (AB SCIEX, Framingham, MA, USA). Proteins were identified using the ProteinPilot Software (AB SCIEX).

### Statistics

All data are expressed as mean ± standard deviation. Two-tailed Student’s t-tests with Bonferroni correction were used for the statistical evaluation.

## Results

### Characterization of a novel ubiquitin ligase RNF183

**Fig 1:**
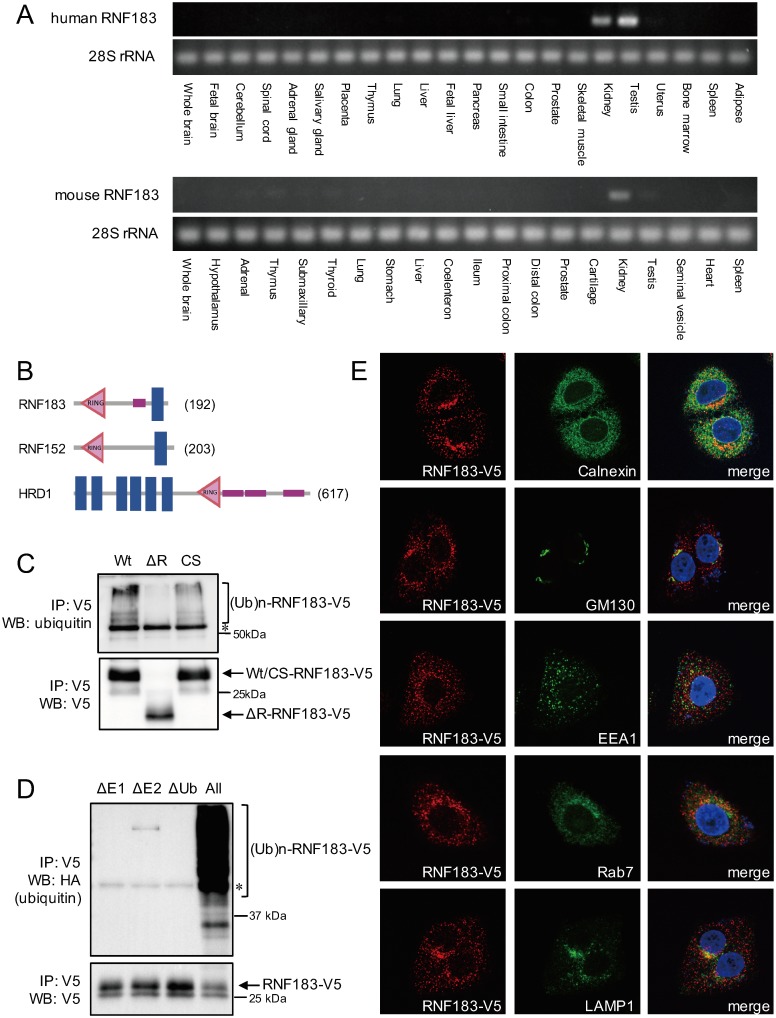
Characterization of the novel ubiquitin ligase RNF183. (A) Expression of RNF183 mRNA in various human and murine tissues. RNF183 mRNA expression in 22 human and 20 murine tissues was analyzed using RT-PCR. (B) Schematic diagrams of the predicted domains of transmembrane ubiquitin ligases. Prediction of domains for E3 ligases were made using SMART [6]. *Blue* box, transmembrane domain; *red* triangle, RING-finger domain; *purple* box, low complexity sequence. The number at *right* indicates the peptide length. (C) *In vitro* auto-ubiquitination of wild type and mutant RNF183. *In vitro* transcribed/translated V5-tagged RNF183 tagged was mixed and incubated with recombinant E1, E2, and HA-ubiquitin. The reaction mixture was immunoprecipitated with an anti-V5 antibody and subjected to Western blotting with anti-polyubiquitin (*upper* panel) and anti-V5 antibodies (*lower* panel). WT, wild type; ΔR, RING-finger domain deletion mutant; CS, Cys13-, and Cys16-to-Ser point mutations in the RING domain; IP, immunoprecipitation; Ub, ubiquitin. Asterisk indicates the immunoglobulin heavy chain. (D) *In vitro* auto-ubiquitination of RNF183 in the absence of each component. (E) Subcellular localization of RNF183. HeLa cells stably transfected with RNF183-V5 (*red*) were subjected to immunofluorescence staining with various antibodies for organelle makers (Calnexin, GM130, EEA1, Rab7 and LAMP1; *green*) and DAPI for nuclear staining (*blue*).

We initially performed RT-PCR using cDNA generated from human and murine tissue RNAs to investigate the distribution patterns of the 37 identified ubiquitin ligases. We identified an uncharacterized kidney-abundant gene, RNF183 (Fig 1A), that encodes a 192-amino-acid protein containing an N-terminal RING-finger domain (C3HC4 type) and C-terminal transmembrane domain (Fig 1B). We further determined that the RNF183 protein is conserved from fish to mammals (S1 Fig).

We next examined the auto-ubiquitination activity of RNF183 *in vitro* to assess the efficacy of this protein as an E3 ubiquitin ligase. *In vitro* transcribed/translated RNF183 was incubated with recombinant ubiquitin-activating enzyme (E1), ubiquitin-conjugating enzyme (E2), and HA-tagged ubiquitin in the presence of ATP. The ubiquitination of immunoprecipitated RNF183 was subsequently detected using an anti-polyubiquitin antibody (Fig 1C, S2 Fig). On Western blots, wild-type (WT) RNF183 appeared as a smear of high molecular weight bands indicative of auto-ubiquitination (Fig 1C, *upper* panel, lane 1). In contrast, the ΔR mutant, which lacks the RING-finger domain, did not exhibit this ubiquitinated band (Fig 1C, *upper* panel, lane 2), whereas the CS mutant, in which consensus cysteines of the RING-finger domain are substituted by serine, exhibited decreased smears (Fig 1C, *upper* panel, lane 3). In addition, the smear band of WT RNF183 disappeared in the absence of E1, E2, or ubiquitin (Fig 1D). Taken together, these data indicate that RNF183 possesses ubiquitin ligase activity.

To determine the subcellular localization of RNF183, we subjected V5-tagged RNF183-expressing HeLa cells to double labeling with V5-tagged RNF183 and endogenous organelle markers (Fig 1D, S3 Fig). RNF183 exhibited a punctate distribution throughout the cell. RNF183 predominantly colocalized with the ER marker protein Calnexin, the *cis*-Golgi marker GM130, and the lysosomal marker LAMP1, and partially colocalized with early endosome marker EEA1 and late endosome marker Rab7 and Rab9. These results were consistent with those from COS1 cells stably expressing V5-tagged RNF183 (S4 Fig).

### RNF183 interacts and colocalizes with Sec16A

**Fig 2:**
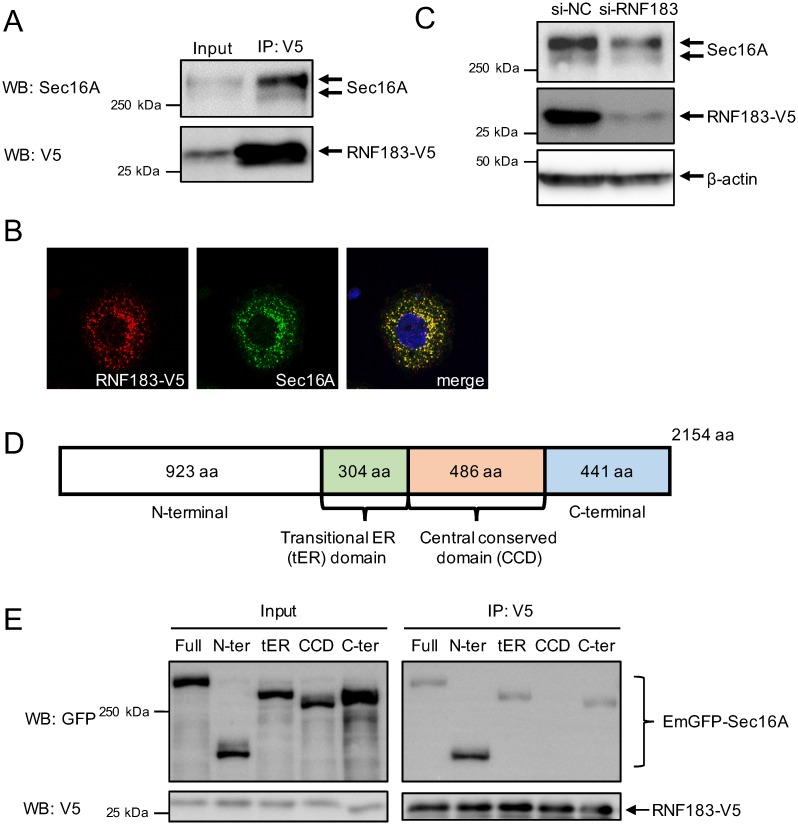
Interaction and colocalization of RNF183 and Sec16A. (A) Interaction of RNF183 and Sec16A. Cell lysates from HEK293 cells stably expressing V5-tagged RNF183 were immunoprecipitated using anti-V5 antibody, and the immune complexes were analyzed by Western blotting with anti-Sec16A (*upper* panel) or anti-V5 (*lower* panel) antibodies. (B) Colocalization of RNF183 with Sec16A. HeLa cells stably transfected with RNF183-V5 were subjected to immunofluorescence staining with anti-Sec16A (*green*) and DAPI (*blue*). (C) Effect of RNF183 knockdown on Sec16A protein. RNF183 expression in HEK293 cells stably transfected with RNF183-V5 was suppressed using siRNA. Endogenous Sec16A protein levels were detected using Western blotting with anti-Sec16A antibody. NC, negative control. (D) Schematic diagram of the predicted domains of Sec16A. (E) Interaction domain of Sec16A with RNF183. Lysates from HEK293 cells stably expressing RNF183 transiently transfected with GFP-tagged full-length Sec16A (Full) or its deletion mutant constructs were subjected to immunoprecipitation with anti-V5 antibody, followed by immunoblotting with anti-GFP antibody.

To identify RNF183 interacting proteins, we performed a mass spectrometric analysis of proteins that were coimmunoprecipitated with RNF183 from HEK293T cells engineered to stably express RNF183. SEC16 Homolog A (Sec16A), which is localized at the ER exit site and participates in the formation of COPII vesicles, was the most frequently detected protein (Table 1). Next, we confirmed the interaction between RNF183 and Sec16A using immunoprecipitation and Western blotting, and found that exogenously expressed RNF183 interacted with endogenous Sec16A (Fig 2A). Additional coimmunostaining of RNF183 and Sec16A revealed a significant overlap of RNF183 and Sec16A (Fig 2B, S4 Fig). To determine whether Sec16A is a substrate for RNF183 ubiquitin ligase, we examined the effect of RNF183 knockdown and overexpression on Sec16A protein expression. A significant decrease in Sec16A protein levels was observed in cells subjected to RNF183 knockdown (Fig 2, S5 Fig); whereas, overexpression of RNF183 did not affect Sec16A protein levels (S6 Fig). *In vitro* ubiquitination assay of Sec16A by RNF183 revealed that RNF183 did not ubiquitinate Sec16A (S7 Fig). These results indicate that Sec16A is not a substrate for RNF183. Sec16A can be divided into four main domains: N-terminal domain, transitional ER (tER) domain, central conserved domain (CCD), and C-terminal domain (Fig 2D). Immunoprecipitation experiment of Sec16A deletion mutants revealed that the CCD domain is responsible for the interaction between RNF183 and Sec16A, since Sec16A lacking the CCD domain was not coimmunoprecipitated with RNF183 (Fig 2E). Furthermore, we examined the colocalization of Sec16A lacking the CCD domain with RNF183. Immunofluorescence imaging revealed that Sec16A lacking the CCD domain did not colocalize with RNF183 (S8 Fig).

**Table 1:** Proteomics analysis of the RNF183 interacting protein.

Cell name	Peptide cover rate (%)	Number of detected peptides	Protein name
HEK293	32.7	24	Protein transport protein Sec16A

### Effect of Sec16A knockdown on RNF183 protein stability

**Fig 3:**
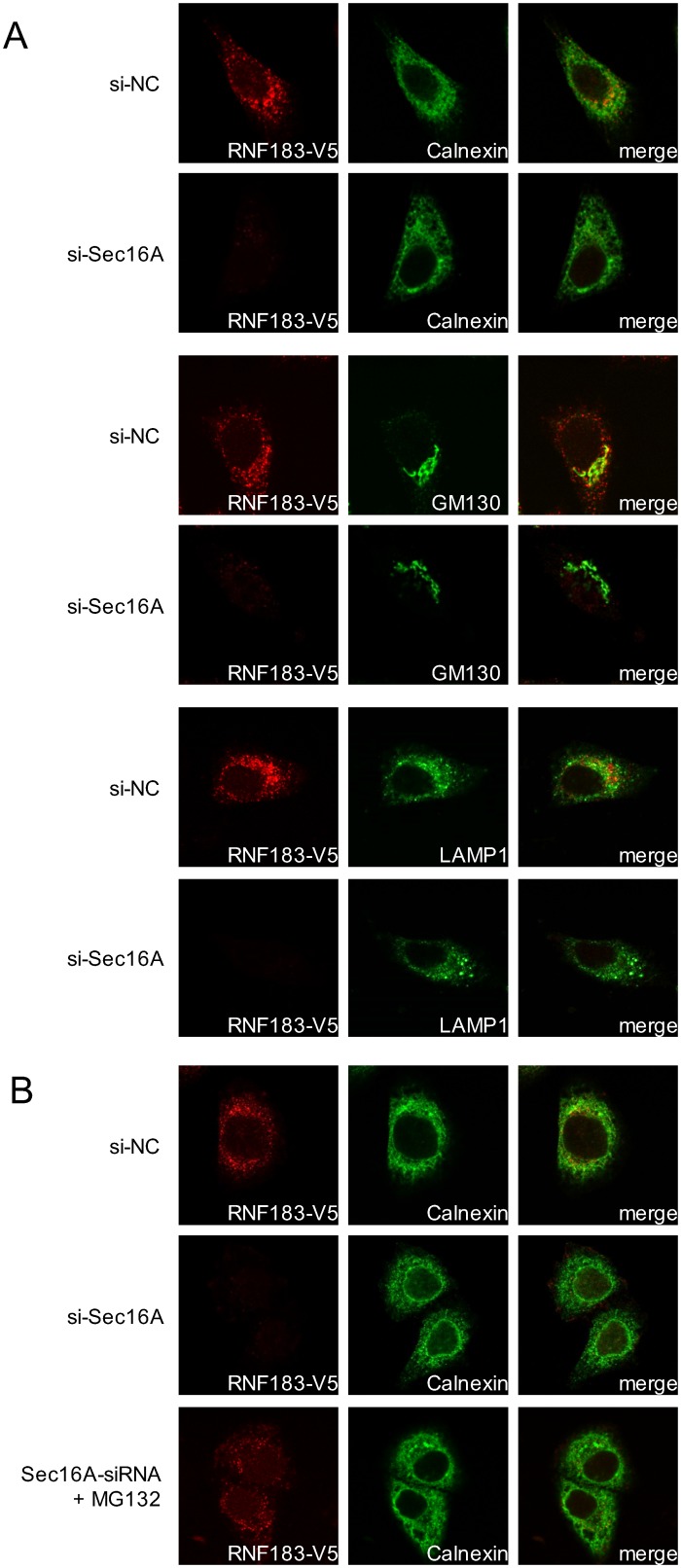
Effects of Sec16 on RNF183 subcellular localization. (A) Effect of Sec16A downregulation on RNF183 subcellular localization. HeLa cells stably expressing RNF183-V5 were transfected with NC (1st, 3rd, 5th panels) or Sec16A (2nd, 4th, 6th panels) siRNAs. At 48 h after transfection, cells were subjected to immunofluorescence staining with anti-V5 (*green*) and anti-calnexin, GM130, or LAMP1 (*red*) antibodies, and DAPI (*blue*). (B) Effect of proteasome inhibition on RNF183 subcellular localization. HeLa cells stably expressing RNF183-V5 were transfected with NC (*top* panels) or Sec16A (*middle* and *bottom* panels) siRNAs. At 36 h after transfection, cells were incubated with (*bottom* panels) or without (*top* and *middle* panels) 10 μM MG132 for 12 h.

We initially evaluated the effect of Sec16A knockdown on RNF183 subcellular localization via immunofluorescence staining to better understand the significance of the interaction between Sec16A and RNF183 (S9 Fig). Notably, RNF183 disappeared from the all organelles in the absence of Sec16A (Fig 3A; 2nd, 4th, 6th panels). However, the expression and ER localization of RNF183 were restored by treatment with the proteasome inhibitor MG132 (Fig 3B, *bottom* panels).

**Fig 4:**
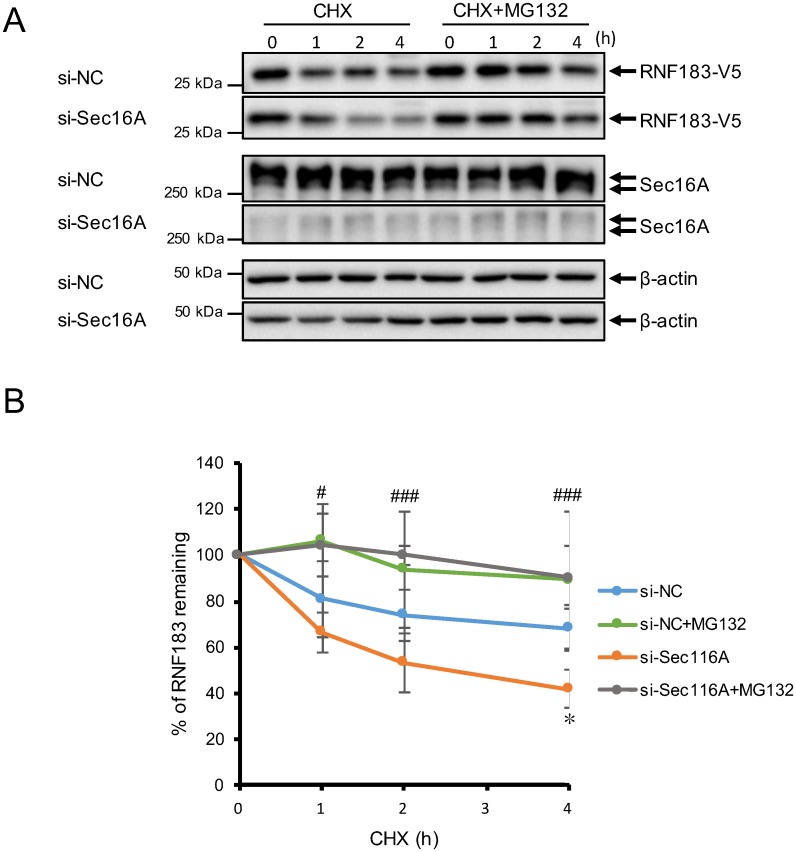
Effects of Sec16 on RNF183 protein stability. (A) Effect of Sec16A downregulation on RNF183 protein stability. HeLa cells stably expressing RNF183-V5 were transfected with NC (1st, 3rd, and 5th panels) or Sec16A (2nd, 4th and 6th panels) siRNA. At 44 h after transfection, cells were treated with 30 μg/ml cycloheximide (CHX) and 10 μM MG132 for the indicated periods. Total cell lysates were analyzed by Western blotting with an anti-V5 (1st and 2nd panels), Sec16A (3rd and 4th panels), and β-actin (5th and 6th panels) antibodies. (B) Quantitative curves of data from (A). RNF183 levels at each time point were plotted relative to the level at time 0 (n = 3). Asterisks represent significant differences (Student’s t test with Bonferroni correction, *p < 0.05; NC vs. Sec16A siRNA; #p < 0.05, ##p < 0.01; ##p < 0.001; Sec16A siRNA vs. Sec16A siRNA + MG132).

Next, we analyzed the effect of Sec16A downregulation on RNF183 protein stability in a cycloheximide chase assay (Fig 4A and 4B). Here, the RNF183 protein levels decreased significantly relative to the negative control at 4 h after the cycloheximide-mediated inhibition of protein synthesis, indicating that RNF183 protein becomes unstable in the absence of Sec16A. We further analyzed the RNF183 protein levels in the treatment with the proteasome inhibitor MG132. RNF183 become much more stable with MG132 in the absence of Sec16A (Fig 4A and 4B). These results suggest that RNF183 cannot dislocate from the ER in the absence of Sec16A, and instead is subject to ERAD-mediated proteasomal degradation.

### Interaction of Sec16A and transmembrane ubiquitin ligases

**Fig 5:**
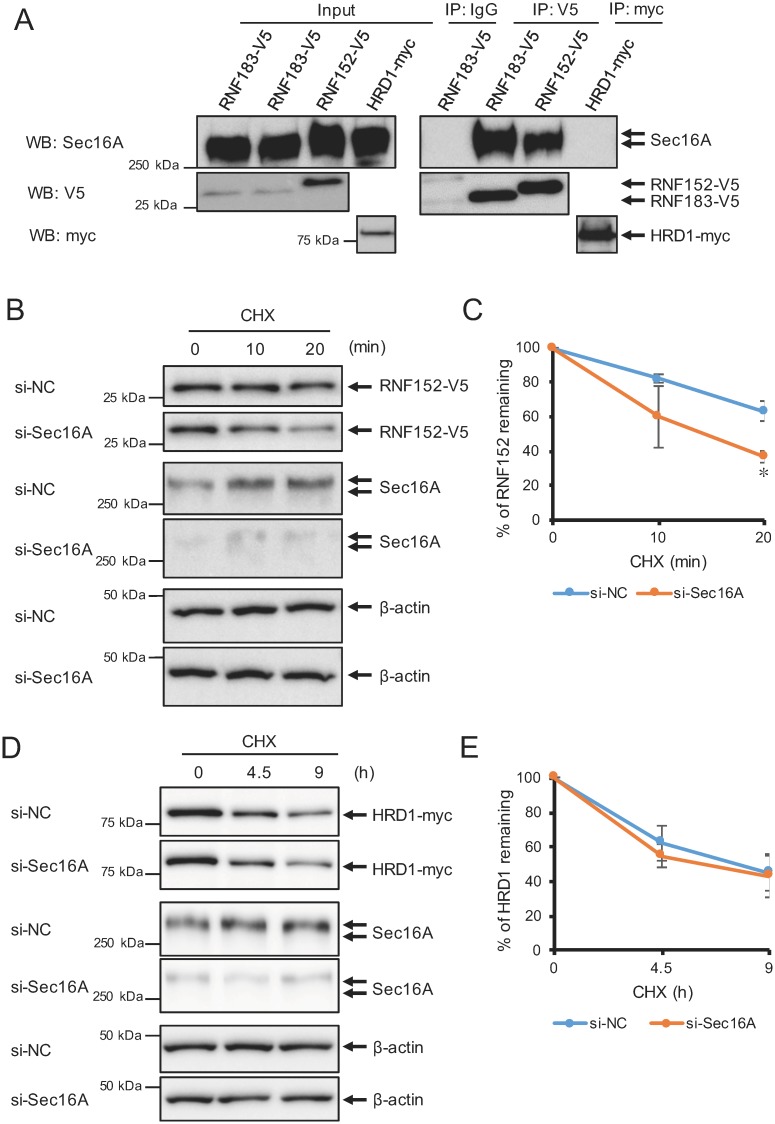
Effects of Sec16 on other ubiquitin ligases. (A) Interactions of RNF152 and HRD1 with Sec16A. Coimmunoprecipitation was performed in HEK293 cells engineered to stably express V5-tagged RNF183, V5-tagged RNF152, or myc-tagged HRD1. Cell lysates were immunoprecipitated with anti-V5 or anti-myc antibodies or normal mouse immunoglobulin G (IgG; negative control). Immune complexes were analyzed by Western blotting with an anti-Sec16A antibody (*top* panel) and anti-V5 (*second* panel) or anti-myc antibodies (*third* panel). (B, D) Effect of Sec16A downregulation on RNF152 and HRD1 protein stability. Stable RNF152-V5- or HRD1-myc-expressing HEK293 cells were transfected with NC or Sec16A siRNA. At 48 h after transfection, cells were subjected to a CHX assay. (C, E) Asterisks represent significant differences (n = 3; Student’s t test with Bonferroni correction, *p < 0.05, ***p < 0.001; NC vs. Sec16A siRNA).

Next, we confirmed the interactions of Sec16A with the ubiquitin ligases RNF152 and HRD1 to investigate the specificity of Sec16A for other transmembrane ubiquitin ligases (Fig 1B). RNF152 has similar structural and lysosomal localization characteristics with RNF183 [7], whereas HRD1 is structurally different from RNF183 and specifically localizes in the ER [5]. Here, we observed an interaction between Sec16A and RNF152, but not between Sec16A and HRD1 (Fig 5A). We additionally investigated the effect of Sec16A knockdown on the stability of RNF152 and HRD1 proteins. We observed a significantly increased RNF152 degradation rate in the absence of Sec16A when compared with the controls (Fig 5B and 5C); in contrast, the HRD1 degradation rate was unaffected by Sec16A knockdown (Fig 5D and 5E). These results suggest that Sec16A can regulate the stability of ubiquitin ligases it interacts with.

## Discussion

RNF183 was recently described as an interacting protein for the cancer-testis antigen, Foetal and Adult Testis Expressed 1 (FATE1), through a yeast two-hybrid screening analysis [8]. Another study demonstrated that RNF183 expression is strongly upregulated in inflamed colon samples from patients with inflammatory bowel disease [9]. In this study, we identified the kidney-abundant ubiquitin ligase in normal human and mouse tissues, suggesting that under normal conditions, expression of this protein is restricted to the kidney but is likely upregulated elsewhere in response to various stressors. Notably, we did not observe the induction or upregulation of RNF183 expression in response to ER stress [3].

According to our immunocytochemical analysis, RNF183 predominantly localizes in the lysosome. Another ubiquitin ligase, RNF152, which has structural similarities with RNF183, also predominantly localizes in the lysosome [7]. A recent study suggested that RNF152 is involved in the regulation of autophagy through the K63-linked ubiquitination of RagA, a positive regulator of mTOR signaling, in the lysosome [10]. Further studies are needed to determine whether RNF183 shares this function with RNF152, given the similar localization pattern. In addition, the identification of RNF183 substrates will facilitate a better understanding of the role of RNF183.

To elucidate the functional roles of RNF183, we performed a proteome analysis to identify RNF183 interacting proteins. Sec16A, a key regulator of COPII vesicle formation, was most frequently identified in this analysis, thus we evaluated its potential as a substrate for RNF183. Although the Sec16A protein level was not elevated by the loss of RNF183 expression, indicating that Sec16A is not a substrate, RNF183 protein expression became unstable in the absence of Sec16A but was restored following treatment with the proteasome inhibitor MG132. These results suggest that RNF183 does not act as ubiquitin ligase in ERAD, but may be degraded by ERAD if it is not transported from the ER. Further analysis identified that RNF152 interacted with Sec16A in a similar manner as RNF183, whereas another transmembrane ubiquitin ligase HRD1, which exhibits different characteristics, did not associate with Sec16A. We propose that Sec16A regulates the stability of ubiquitin ligases it interacts with, those structurally similar to RNF183, and thus promotes the transportation of its interacting proteins from the ER to the Golgi via COPII vesicles.

Sec16A expression is induced by ER stress in a response mechanism conserved from yeast to mammalian cells [11, 12]. In addition, RNF183 expression is increased in response to Sec16A overexpression. Therefore, we speculated that ER stress might upregulate RNF183 protein expression via the increased expression of Sec16A. Unexpectedly, however, the RNF183 protein level decreased under conditions of ER stress (S11 Fig). These results suggest that under severe ER stress conditions, such as those induced by tunicamycin, RNF183 protein folding is impaired, thus blocking its interaction with Sec16A and promoting its degradation by ERAD. Therefore, to understand the role of Sec16A during ER stress, it may be necessary to examine the stability and transportation of endogenous RNF183 under near-physiological conditions of ER stress.

Previous reports have suggested a Sec16A regulatory pathway involving the autophagy initiators ULK1 and ULK2, which are in turn regulated by kinases such as mTORC1 and AMPK. The phosphorylation of Sec16A by ULKs is an essential step in ER-to-Golgi trafficking [13]. The activation of Sec16A by kinases such as ULKs might be required prior to the translocation of RNF183. Furthermore, a recent report indicated that Sec16A interacts with the GTPase domain of LRRK2, a protein produced by the causative gene for Parkinson’s disease, and observed that LRRK2 regulates the function of Sec16A [14]. LRRK2 is expressed in the kidney, and LRRK2 knockout mice have been found to exhibit kidney-specific lysosomal abnormalities and enhanced autophagy [15]. Therefore, it is important to determine the involvement of LRRK2-mediated Sec16A regulation in the transportation of RNF183 and RNF152 and to elucidate the functions of these proteins in the lysosome, particularly with regard to autophagy.

In conclusion, the novel, kidney-specific transmembrane ubiquitin ligase RNF183 is not involved in ERAD. However, Sec16A, which regulates COPII-vesicle formation, mediates the stabilization and ER export of RNF183.

## Supporting information

S1 Fig Phylogenetic tree for RNF183. The RNF183 family gene tree was constructed using the TreeFam database (http://www.treefam.org).
(TIFF)

S2 Fig *In vitro* auto-ubiquitination of wild type and mutant RNF183. *In vitro* transcribed/translated V5-tagged RNF183 tagged was mixed and incubated with recombinant E1, E2, and HA-ubiquitin. The reaction mixture was immunoprecipitated with an anti-V5 antibody and subjected to Western blotting with anti-V5 antibodies. WT, wild type; ΔR, RING-finger domain deletion mutant; CS, Cys13-, and Cys16-to-Ser point mutations in the RING domain; IP, immunoprecipitation; Ub, ubiquitin.
(TIFF)

S3 Fig Subcellular localization of RNF183 in HeLa cells. HeLa cells stably transfected with RNF183-V5 (*red*) were subjected to immunofluorescence staining with various antibodies for organelle makers (Rab9, Golgin-97, COX IV, and N-K ATPase; *green*) and DAPI for nuclear staining (*blue*).
(TIFF)

S4 Fig Subcellular localization of RNF183 in COS-1 cells. COS-1 cells stably transfected with RNF183-V5 (*red*) were subjected to immunofluorescence staining with various antibodies for organelle makers (Rab9, Golgin-97, COX IV, and N-K ATPase; *green*) and DAPI for nuclear staining (*blue*).
(TIFF)

S5 Fig Quantitative data from Fig 2C. RNF183 levels were compared between si-Nontarget control (NC) and si-Sec16A (n = 3). Asterisks represent significant differences (Student’s *t* test, *p < .05; NC vs. Sec16A siRNA).
(TIFF)

S6 Fig Effect of RNF183 overexpression on Sec16A protein. HEK293 cells were transfected with emerald-green fluorescent protein (EmGFP)-RNF183 and were subjected to Western blotting with anti-GFP antibody for EmGFP-RNF183.
(TIFF)

S7 Fig *In vitro* ubiquitination of Sec16 by RNF183. *In vitro* transcribed/translated V5-tagged RNF183 was mixed and incubated with *in vitro* transcribed/translated Sec16A and recombinant E1, E2, and HA-ubiquitin. The reaction mixture was immunoprecipitated with an anti-HA antibody and subjected to Western blotting with anti-Sec16A antibodies (*left* panel). *Right* panel is auto-ubiquitination of RNF183 as a control.
(TIFF)

S8 Fig Colocalization of RNF183 and ΔCCD-Sec16A. HeLa cells stably expressing RNF183-V5 were transfected with EmGFP-Sec16A lacking the CCD domain. At 48 h after transfection, cells were subjected to immunofluorescence staining with anti-V5 (*read*) antibody. emerald-green fluorescent protein (EmGFP) (*green*) and DAPI (*blue*).
(TIFF)

S9 Fig Knockdown efficiency of Sec16A. HeLa cells stably expressing RNF183-V5 were transfected with NC (*upper* panels) or Sec16A (*lower* panels) siRNAs. At 48 h after transfection, cells were subjected to immunofluorescence staining with anti-V5 (*read*) and anti-Sec16A (*green*) antibodies, and DAPI (*blue*).
(TIFF)

S10 Fig Full blotting image of Fig 5A. (TIFF)

S11 Fig Effect of ER stress on RNF183 protein levels. HK-2 cells stably expressing RNF183-V5 were treated with thapsigargin and tunicamycin.
(TIFF)

S1 Table Primer sets used for RT-PCR. (PDF)
