# Supplementary figures and images for: Sec16A, a key protein in COPII vesicle formation, regulates the stability and localization of the novel ubiquitin ligase RNF183
Source: PLoS One. 2018 Jan 4;13(1):e0190407. doi: 10.1371/journal.pone.0190407 (PMC5754088; doi:10.1371/journal.pone.0190407)

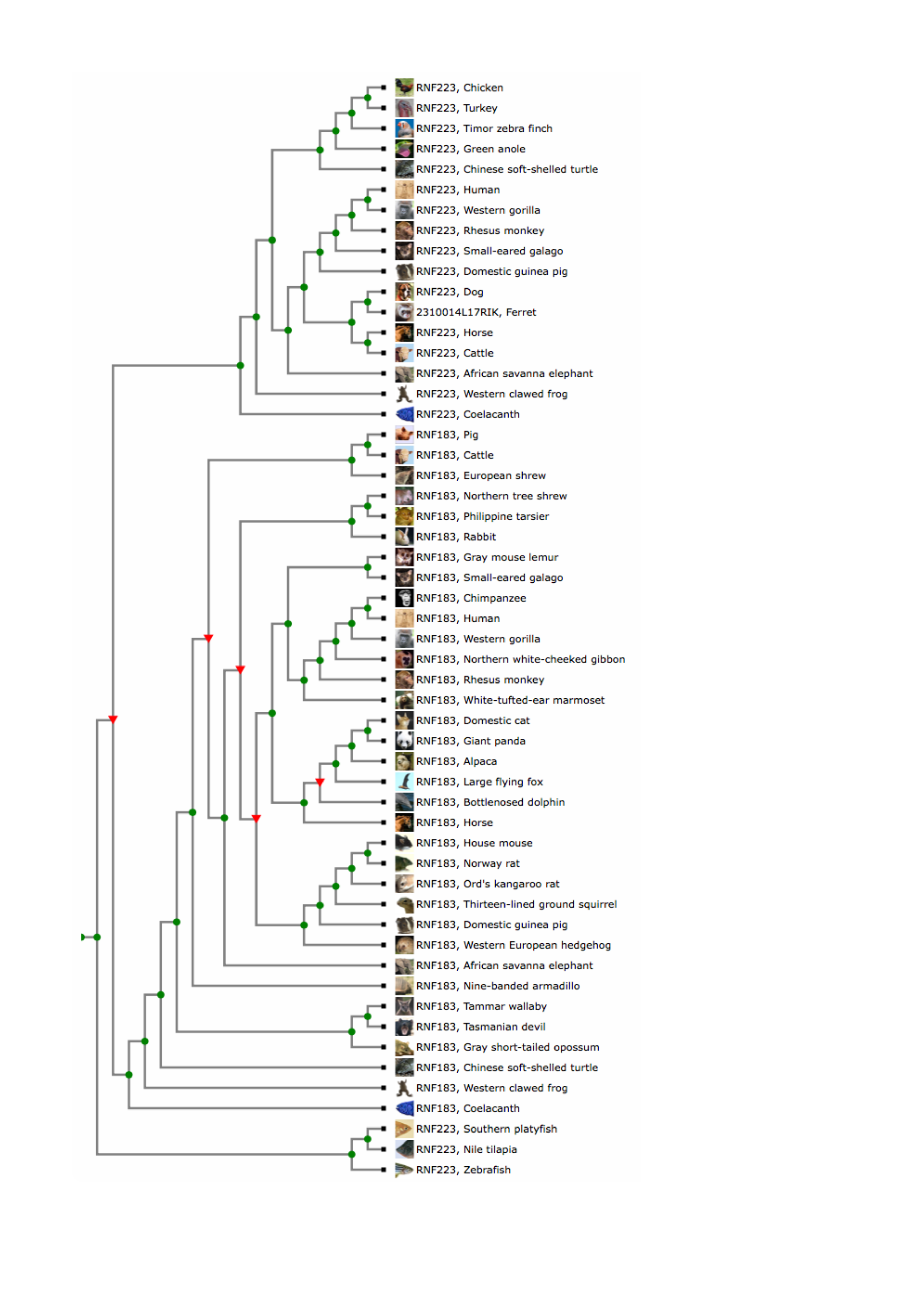

Supplement: S1 Fig — The RNF183 family gene tree was constructed using the TreeFam database (http://www.treefam.org). (TIFF) [file pone.0190407.s001.tiff]

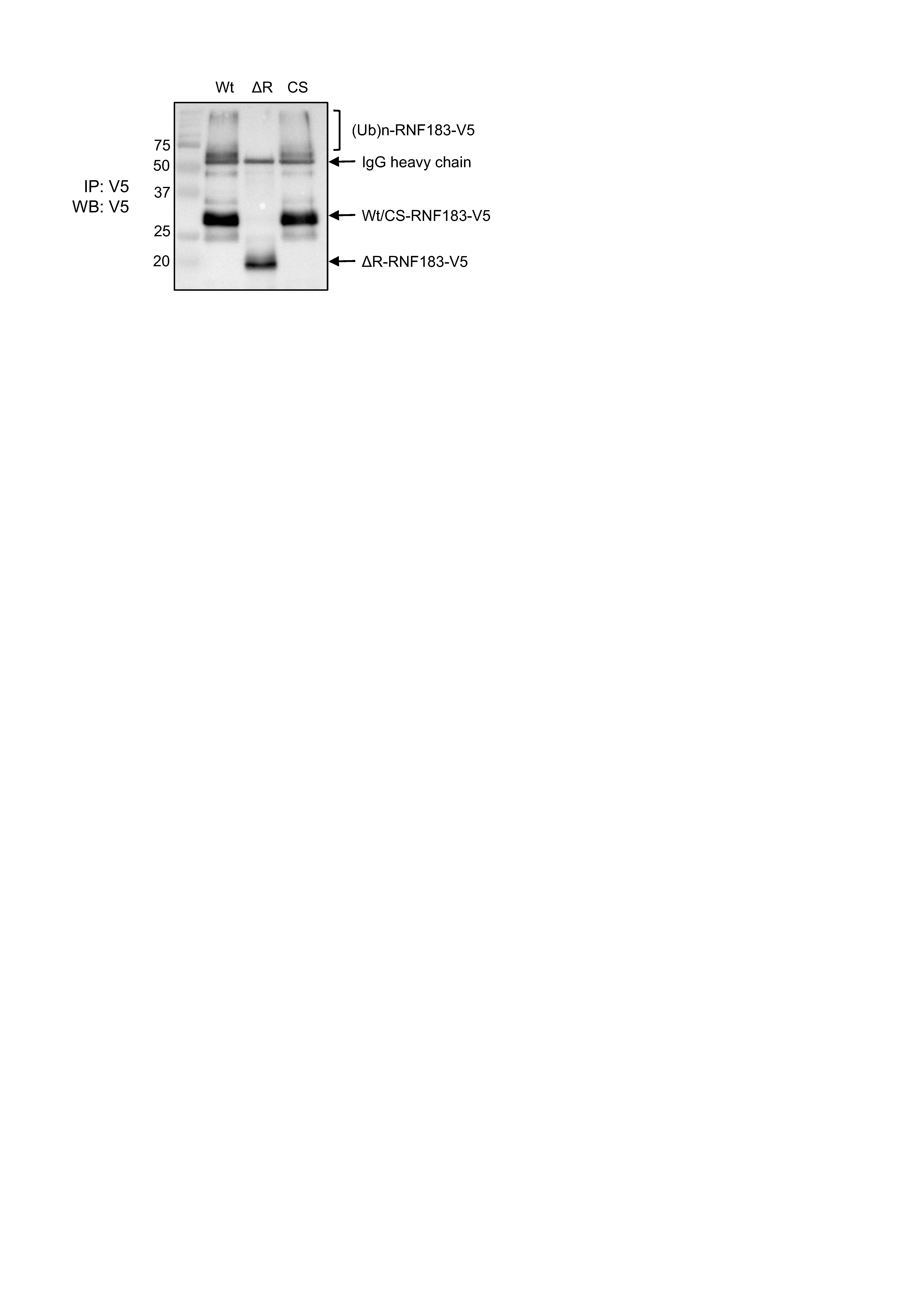

Supplement: S2 Fig — In vitro transcribed/translated V5-tagged RNF183 tagged was mixed and incubated with recombinant E1, E2, and HA-ubiquitin. The reaction mixture was immunoprecipitated with an anti-V5 antibody and subjected to Western blotting with anti-V5 antibodies. WT, wild type; ΔR, RING-finger domain deletion mutant; CS, Cys13-, and Cys16-to-Ser point mutations in the RING domain; IP, immunoprecipitation; Ub, ubiquitin. (TIFF) [file pone.0190407.s002.tiff]

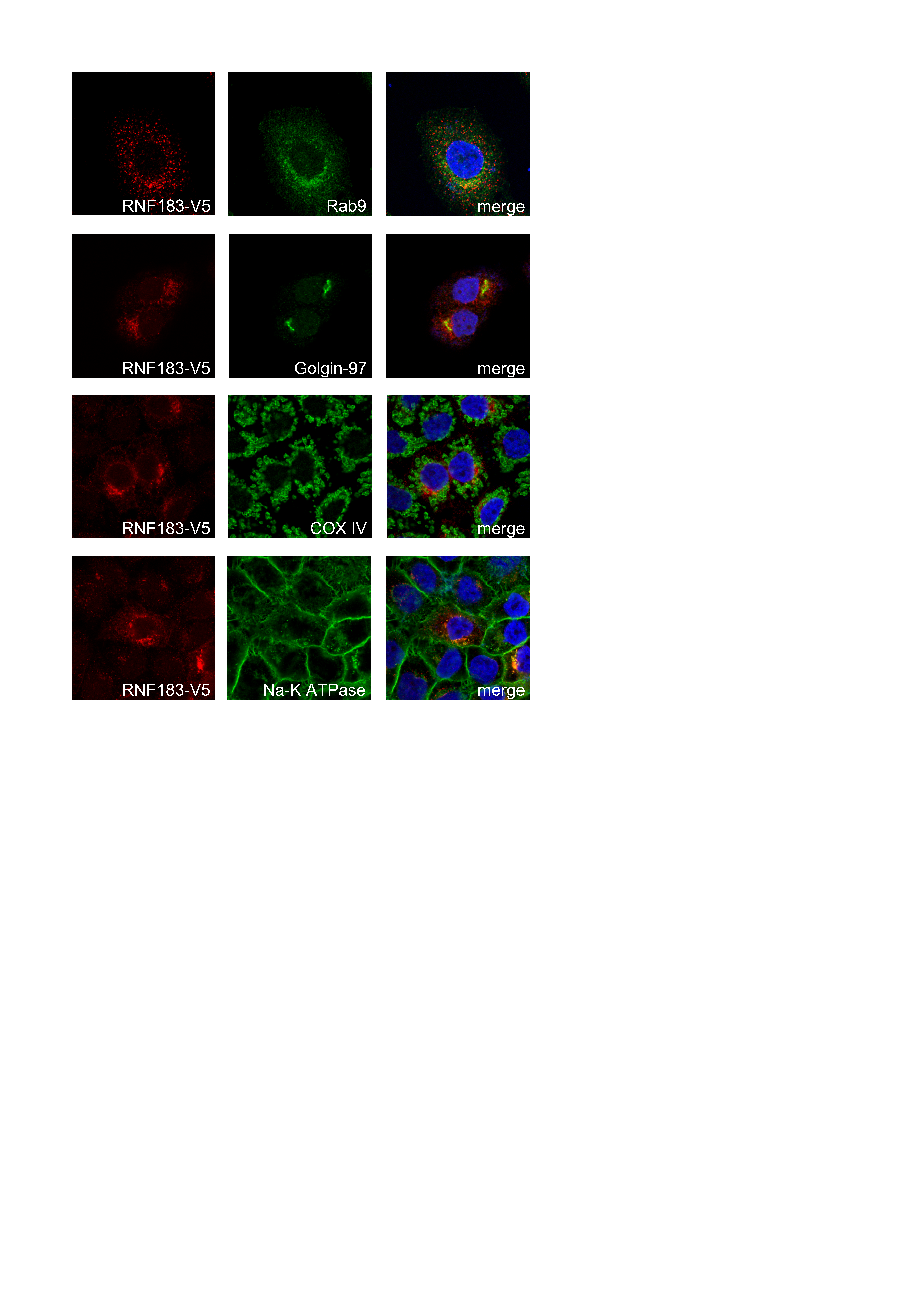

Supplement: S3 Fig — HeLa cells stably transfected with RNF183-V5 (red) were subjected to immunofluorescence staining with various antibodies for organelle makers (Rab9, Golgin-97, COX IV, and N-K ATPase; green) and DAPI for nuclear staining (blue). (TIFF) [file pone.0190407.s003.tiff]

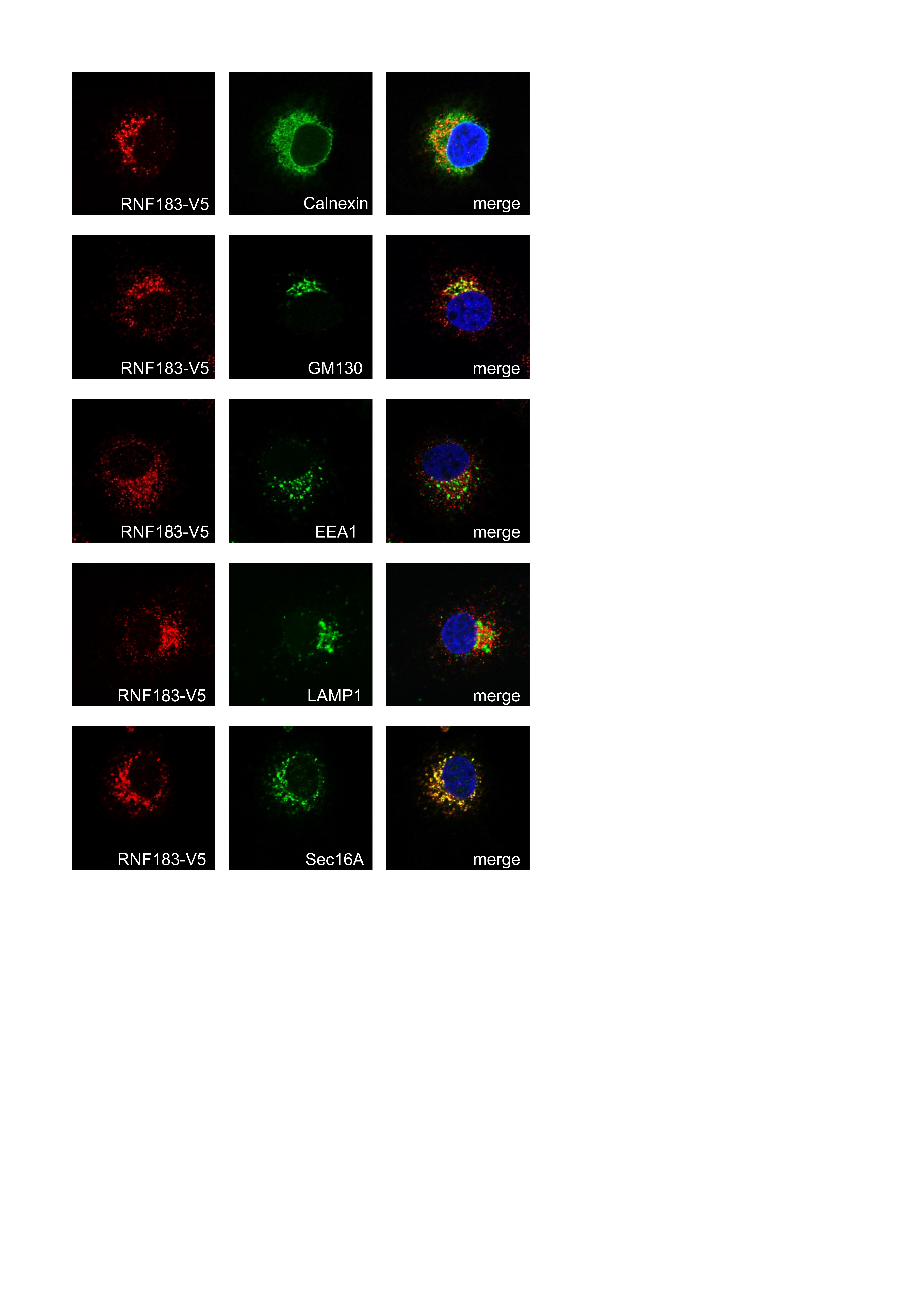

Supplement: S4 Fig — COS-1 cells stably transfected with RNF183-V5 (red) were subjected to immunofluorescence staining with various antibodies for organelle makers (Rab9, Golgin-97, COX IV, and N-K ATPase; green) and DAPI for nuclear staining (blue). (TIFF) [file pone.0190407.s004.tiff]

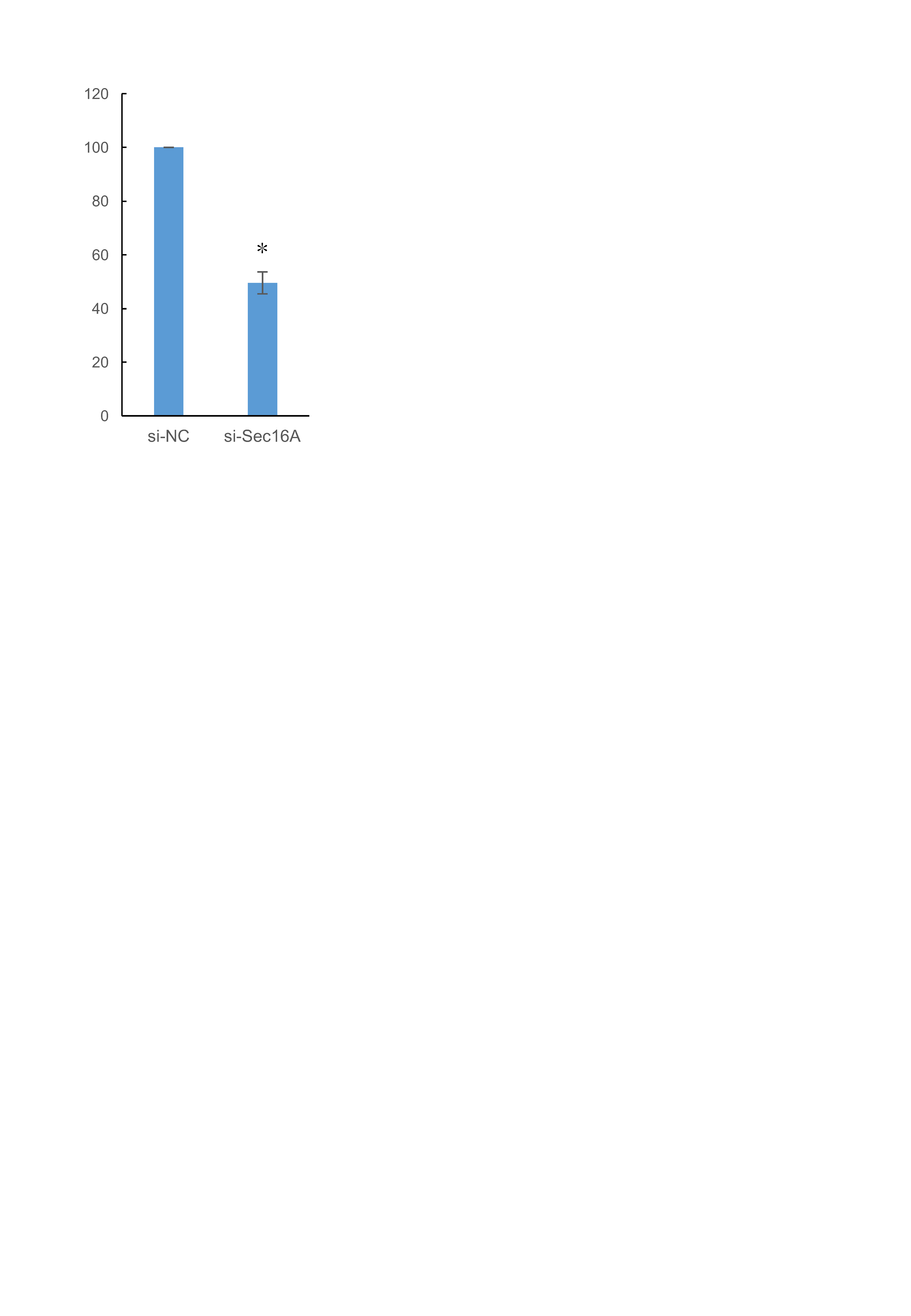

Supplement: S5 Fig — RNF183 levels were compared between si-Nontarget control (NC) and si-Sec16A (n = 3). Asterisks represent significant differences (Student’s t test, *p < .05; NC vs. Sec16A siRNA). (TIFF) [file pone.0190407.s005.tiff]

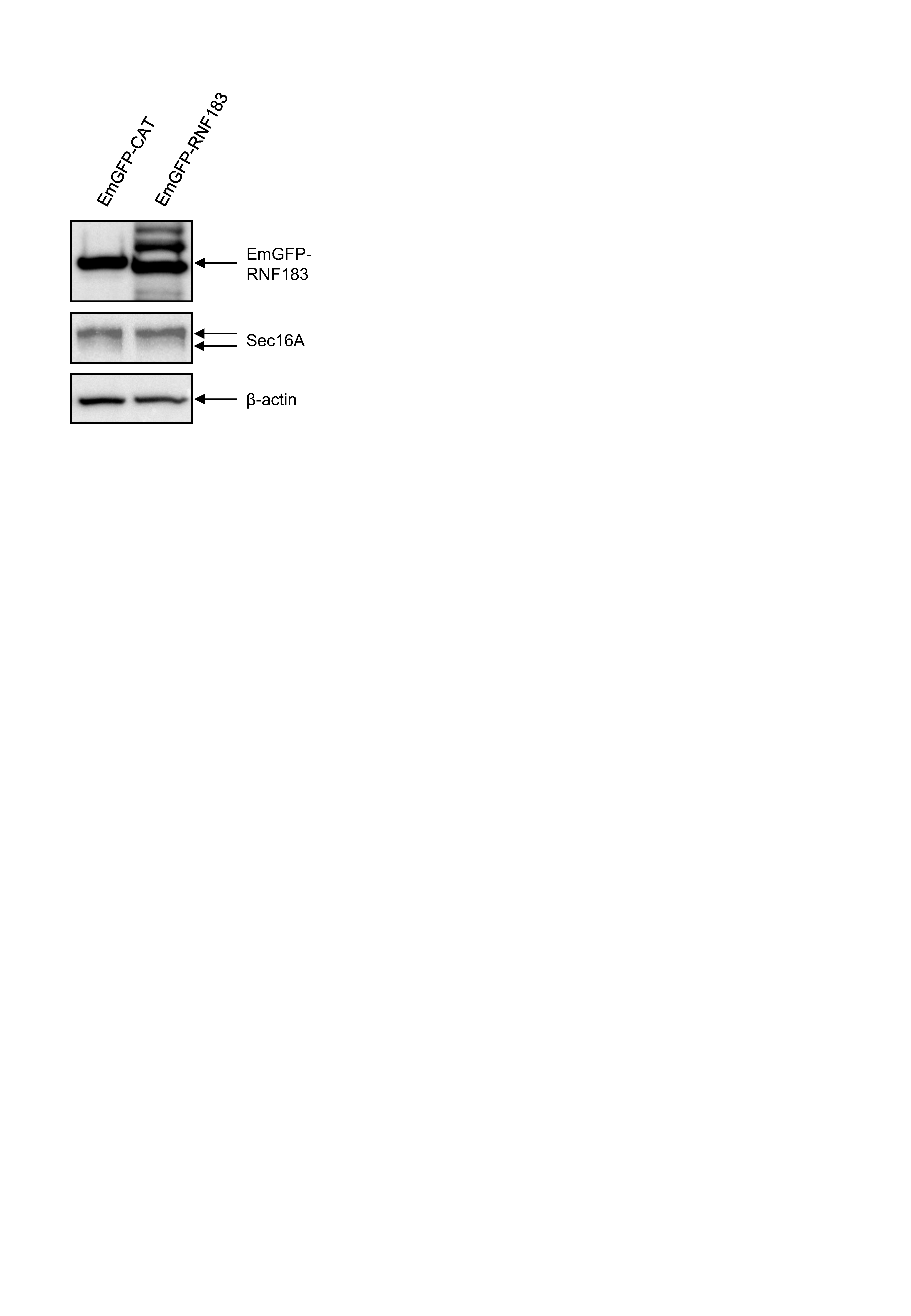

Supplement: S6 Fig — HEK293 cells were transfected with emerald-green fluorescent protein (EmGFP)-RNF183 and were subjected to Western blotting with anti-GFP antibody for EmGFP-RNF183. (TIFF) [file pone.0190407.s006.tiff]

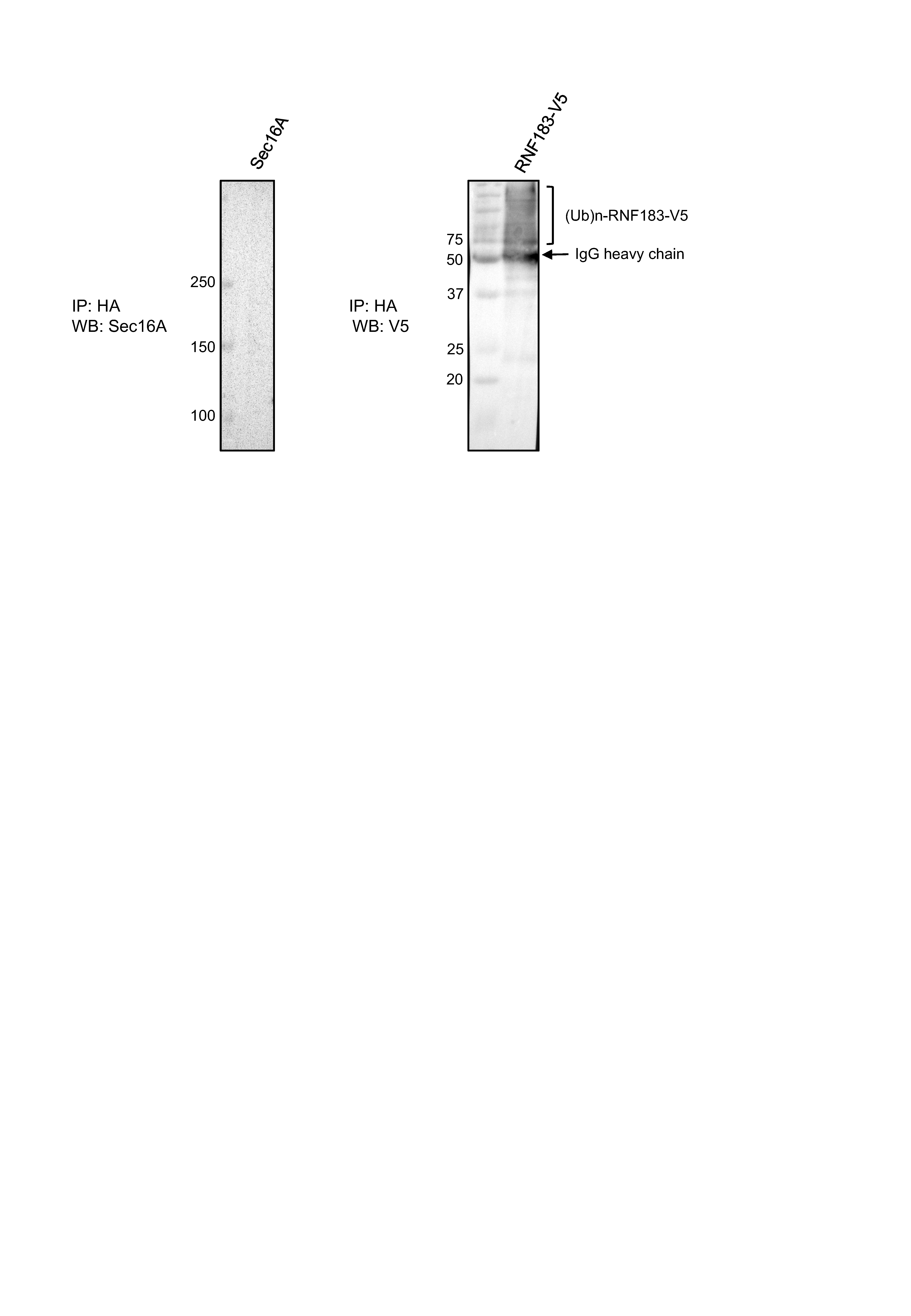

Supplement: S7 Fig — In vitro transcribed/translated V5-tagged RNF183 was mixed and incubated with in vitro transcribed/translated Sec16A and recombinant E1, E2, and HA-ubiquitin. The reaction mixture was immunoprecipitated with an anti-HA antibody and subjected to Western blotting with anti-Sec16A antibodies (left panel). Right panel is auto-ubiquitination of RNF183 as a control. (TIFF) [file pone.0190407.s007.tiff]

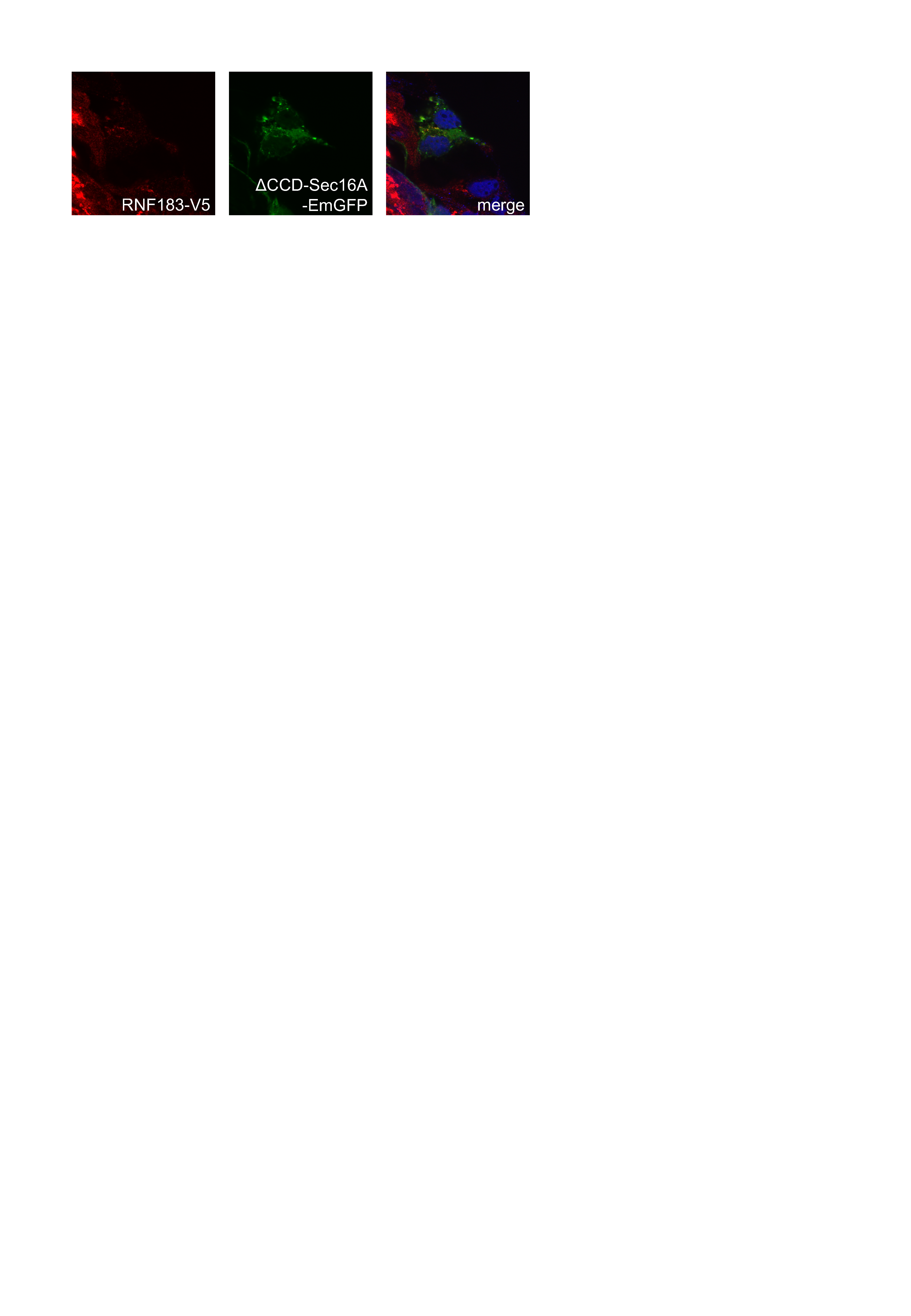

Supplement: S8 Fig — HeLa cells stably expressing RNF183-V5 were transfected with EmGFP-Sec16A lacking the CCD domain. At 48 h after transfection, cells were subjected to immunofluorescence staining with anti-V5 (read) antibody. emerald-green fluorescent protein (EmGFP) (green) and DAPI (blue). (TIFF) [file pone.0190407.s008.tiff]

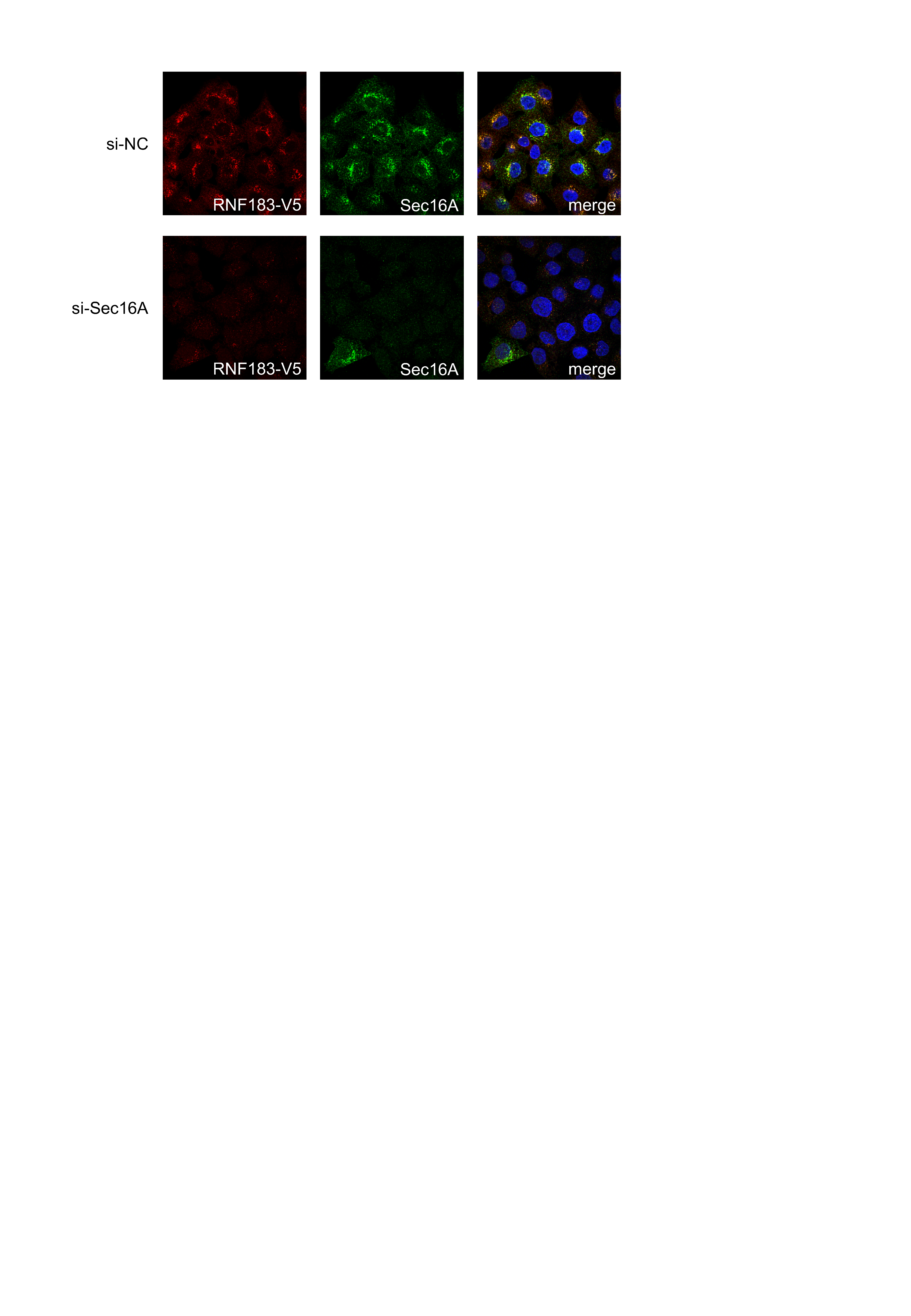

Supplement: S9 Fig — HeLa cells stably expressing RNF183-V5 were transfected with NC (upper panels) or Sec16A (lower panels) siRNAs. At 48 h after transfection, cells were subjected to immunofluorescence staining with anti-V5 (read) and anti-Sec16A (green) antibodies, and DAPI (blue). (TIFF) [file pone.0190407.s009.tiff]

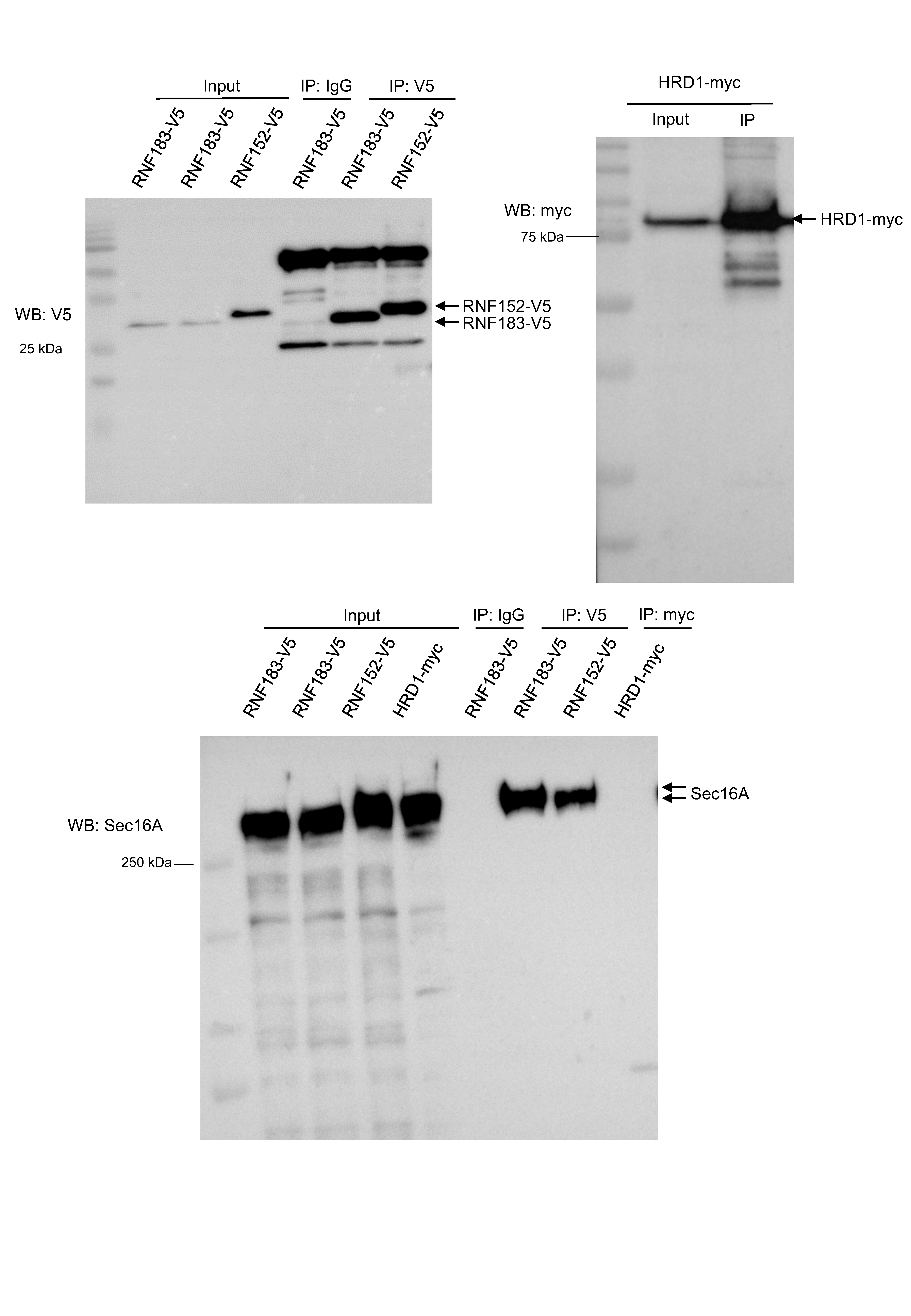

Supplement: S10 Fig — (TIFF) [file pone.0190407.s010.tiff]

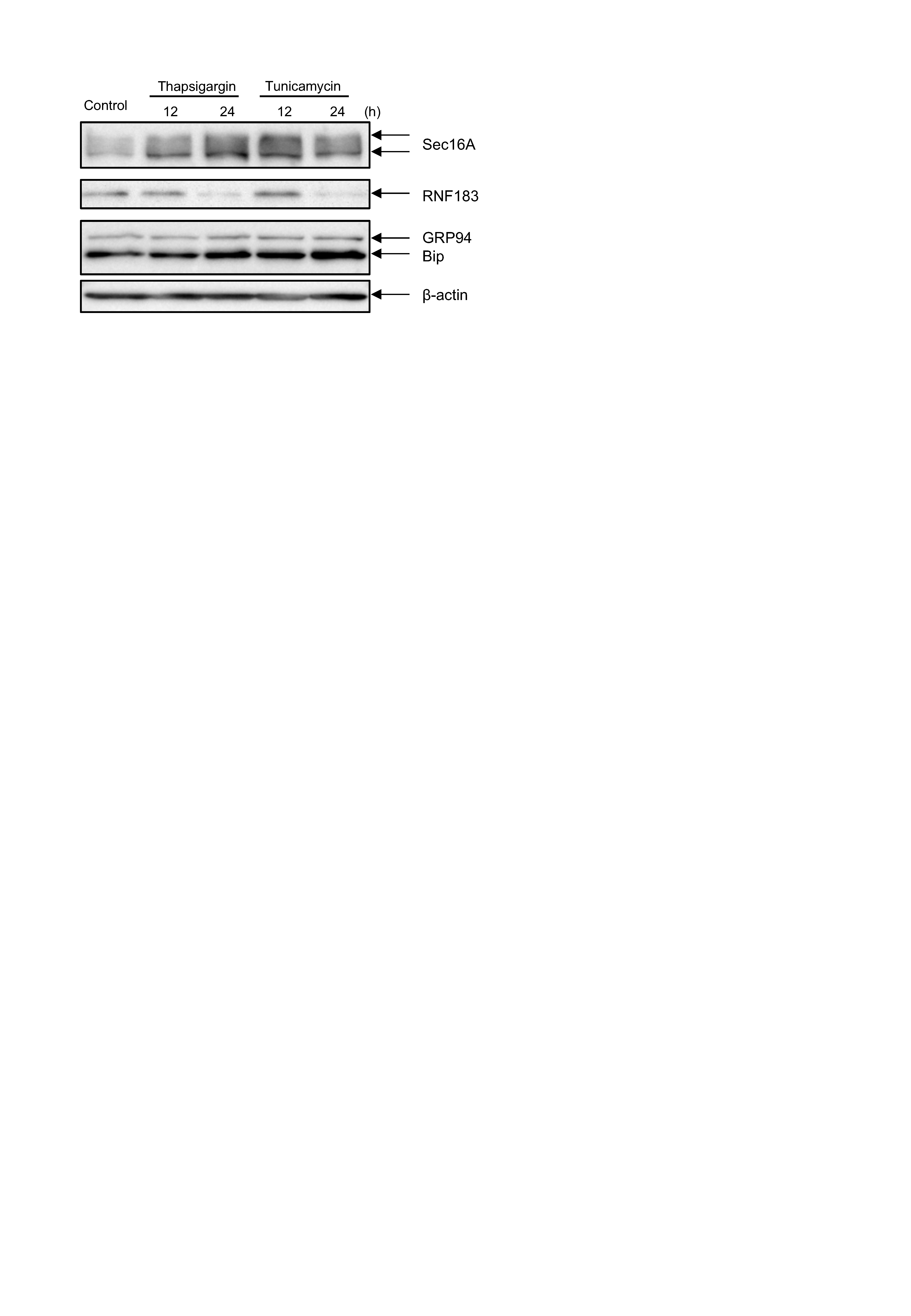

Supplement: S11 Fig — HK-2 cells stably expressing RNF183-V5 were treated with thapsigargin and tunicamycin. (TIFF) [file pone.0190407.s011.tiff]
