# Supplementary material for: Sec16A, a key protein in COPII vesicle formation, regulates the stability and localization of the novel ubiquitin ligase RNF183
Source: PLoS One. 2018 Jan 4;13(1):e0190407. doi: 10.1371/journal.pone.0190407 (PMC5754088; doi:10.1371/journal.pone.0190407)
Supplement: S1 Table — (PDF) [file pone.0190407.s012.pdf]

Gene	Annealing temperature	Cycle	Forward primer	Reverse primer
human RNF183	61.7	30	5’-AGTAGTCTGCCTGACCACC-3’	5’-ATGGCCTTCCAGGATGACA-3’
mouse RNF183	60.0	28	5’-GACCAGCCCAAGAGCCGCTA-3’	5’-CCCCAAAAGAACTGCTTAGTCCA-3’
human & mouse 28S rRNA	55.0	18	5’-TTGAAAATCCGGGGGAGAG-3’	5’-ACATTGTTCCAACATGCCAG-3’
